# Palladin promotes cancer stem cell‐like properties in lung cancer by activating Wnt/Β‐Catenin signaling

**DOI:** 10.1002/cam4.5192

**Published:** 2022-09-01

**Authors:** Xiong Shu, Meng Chen, Shi‐Ya Liu, Long Yu, Li‐Xin Sun, Li‐Chao Sun, Yu‐Liang Ran

**Affiliations:** ^1^ Laboratory of Molecular Orthopaedics Beijing Research Institute of Orthopaedics and Traumatology, Beijing JiShuiTan Hospital Beijing P. R. China; ^2^ State Key Laboratory of Molecular Oncology National Cancer Center/National Clinical Research Center for Cancer/Cancer Hospital, Chinese Academy of Medical Sciences and Peking Union Medical College Beijing P. R. China

**Keywords:** cancer stem cells, lung cancer, Palladin, PALLD, stemness, Wnt/β‐catenin signaling

## Abstract

**Background:**

Cancer stem cells (CSCs) are responsible for drug resistance, cancer relapse, and metastasis. Here, we report the first analysis of Palladin expression and its impacts on stem cell‐like properties in lung cancer.

**Methods:**

Tissue microarrays were used to investigate Palladin expression and its association with prognosis. Immunofluorescence (IF), flow fluorescence assay, and Western blot were performed to detect Palladin expression in 6 NSCLC cell lines. Cell phenotypes and drug resistance were evaluated. Xenograft models were constructed to confirm the role of Palladin in vivo.

**Results:**

By using the tissue microarrays, Palladin was identified to be highly expressed in the cytoplasm, specifically in the cytomembrane of NSCLC, and its high expression is associated with poor prognosis. Palladin is widely expressed and enriched in the sphere cells. The in vitro and in vivo studies showed that Palladin promoted stem cell‐like properties, including cell viability, invasion, migration, self‐renewal abilities, taxol resistance, and tumorigenicity. Western blot revealed that Palladin promoted the accumulation of β‐catenin and activated Wnt/β‐catenin signaling. Tissue microarrays analysis further confirmed the positive correlation between Palladin and β‐catenin. Wnt/β‐catenin pathway inhibitor blocked the Palladin‐induced enhancement of sphere‐forming.

**Conclusions:**

Palladin might act as an oncogene by promoting CSCs‐like properties and tumorigenicity of NSCLC cells via the Wnt/β‐catenin signaling pathway. Besides, Palladin was identified to have the potential as a cell surface marker for LCSCs identification. These findings provide a possible target for developing putative agents targeted to LCSCs.

## Graphical abstract

Palladin was identified to be specifically expressed in the cytomembrane of patients with NSCLC, suggesting its potential as a cell surface marker for LCSCs identification. More importantly, we demonstrated that Palladin might serve as a tumor oncogene and promote the biological properties of LCSCs, possibly by activating the Wnt/β‐catenin signaling in NSCLC for the first time. The elucidation of this new molecular mechanism for Palladin and Wnt/β‐catenin signaling activation is conducive to understand the pathogenesis of NSCLC, providing a novel possibility of the development of novel therapeutic target for CSCs targeted therapy.

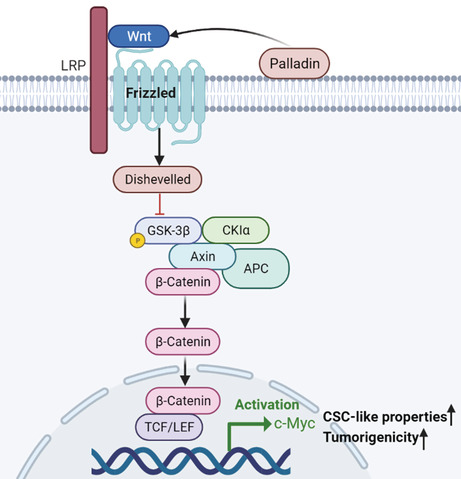

## 1 INTRODUCTION

Stem cell biology has been proposed and come of age over the past decades.^1^ The cancer stem cells (CSCs) are self‐renewable cell types that are identified in most tumors.^2^ Accumulating evidence has demonstrated that CSCs contribute to tumor development, relapse, and metastasis due in part to their intrinsic self‐renewal and tumorigenic properties.^3^ Accordingly, in recent years, therapies that target the CSCs are emerging, even maybe the next avenue of cancer treatment.^4^

Lung cancer remains the leading cause of cancer‐related deaths worldwide in spite of the recent breakthroughs in immunotherapy.^5^ More recently, the widely embraced CSC concept has also been applied to lung cancer.^6^ It is worth noting that several cell surface markers for CSCs identification, such as ABCG2, ALDH, EpCAM, CD133, and CD44, have been widely verified,^7^ but they are not always reliable as certain markers might not be specific in targeting the CSCs of lung cancer (LCSCs).^8^ However, CSCs are often inherently partially resistant to some standard therapies and mediate tumor recurrence.^9^ Thus, although successfully demonstrated in preclinical models, many putative agents targeted at LCSCs have failed in clinical trials.^10^ Currently, understanding the mechanisms and novel targets that regulate LCSCs stemness properties is an urgent need for providing better therapeutic rationales for novel anticancer therapeutics.

Palladin is a member of the actin‐associated proteins family and is highly expressed in multiple tumor cells, including stomach, colon, breast, and pancreas cancers.^11^, ^12^ Notably, due to its role in cell assembly and maintenance, Palladin regulates actin cytoskeleton organization and adhesion formation,^13^ and in turn, contributes to the invasive and migratory nature of cancer metastatic cells.^14^, ^15^, ^16^ Given the central role of CSCs in tumor metastasis,^17^ we assumed that Palladin might be in part responsible for the metastatic and tumorigenic properties of CSCs. Although the contribution of Palladin to the invasion of various cancers, such as breast, and pancreas cancer, has been demonstrated,^16^ its function and mechanism have thus far not been investigated in lung cancer, especially in LCSCs. Wnt/β‐catenin signaling is one of the crucial developmental signaling pathways for CSCs homeostasis and function.^18^ Therefore, the present study was designed to explore whether and how Palladin affects the stemness properties of NSCLC cells by Wnt/β‐catenin signaling.

## 2 MATERIALS AND METHODS

### 2.1 Tissue microarray

Two human lung cancer tissue microarrays were obtained from Shanghai Xinchao Biotechnology. Lung squamous carcinoma and adenocarcinoma microarray contained 90 and 96 pairs of tumors and matched adjacent tissues, respectively.

### 2.2 Cell lines and culture

Human NSCLC cell lines NCI‐H1299, NCI‐H460, GLC‐82, and A549 were preserved at the Cell and Molecular Biology Laboratory, Cancer Institute, Chinese Academy of Medical Sciences. Cell lines SPCA‐1 and NCI‐H226 were obtained from the Shanghai Cell Bank of the Chinese Academy of Sciences. All cell lines were grown in Roswell Park Memorial Institute‐1640 medium containing 10% fetal bovine serum in a 37°C incubator with 5% CO_2_. Serum‐free suspension culture was performed to enrich the sphere cells of various cell lines.

### 2.3 IF

A standard IF staining protocol was conducted, respectively, in live and fixed cells to measure the Palladin expression. Primary antibody against Palladin (Cat No. MA5‐16141, Invitrogen) and secondary antibody prelabeled with DyLight 488 (green, Cat No. 711–486‐152, Jackson Immunoresearch) were used in this study. The nuclear were stained with 4,6‐diamidino‐2‐phenylindole (DAPI). The fluorescent images were acquired through fluorescence microscopy (Nikon).

### 2.4 Flow fluorescence assay

Expression of Palladin in the surface of parental or sphere cells was detected by flow fluorescence assay. In brief, cells were digested with trypsin (0.25%) and incubated with a primary antibody against Palladin (Cat No. MA5‐16141, Invitrogen) followed by a secondary antibody conjugated to DyLight 488 (Cat. No. 711–486‐152, Jackson Immunoresearch). Labeled cells were analyzed using an AccuriC6cytometer (BD Biosciences).

### 2.5 Lentiviral vector transduction

The knockdown and overexpression of Palladin were induced by a lentiviral vector. Short hairpin RNAs (shRNAs) targeting human Palladin (#1 CCAAAGAAGGCCAGTAGAA; #2 CGAGGTTAACATACGAAGA) and a scrambled (control) shRNA were cloned into the lentiviral vector pLKD‐CMV‐G&PP‐U6‐shRNA. Palladin (CATAGCGTAAAAGGAGCAACA) were cloned into the lentiviral vector pLOV‐EF1a‐blasticidin‐CMV‐EGFP‐P2A‐3FLAG. The preparation of expression plasmids and lentiviruses encoding shRNA plasmids and titer detection was provided by ObioTechnology (Shanghai). Cells were incubated with lentiviral vectors at an appropriate multiplicity of infection (MOI) in the presence of Polybrene (5 μg/ml; Sigma).

### 2.6 Sphere formation assay

Sphere cells were collected by gentle centrifugation and dissociated with trypsin. The spheres (500 cells/well) were seeded into a low attachment plate containing serum‐free DMEM/F12 medium, 0.8% methylcellulose, 20 ng/ml epidermal growth factor, 10 ng/ml leukemia inhibitory factor, 20 ng/ml basic fibroblast growth factor, and B27 (1:50) factors. The medium was changed every 3 days; after 14‐day incubation, the formed spheres were imaged and counted under an inverted microscope (Nikon).

### 2.7 Cell viability and drug resistance assay

Cell viability and drug resistance to taxol were evaluated using the Cell Counting Kit‐8 (CCK‐8) assay. Briefly, cells were seeded on a 96‐well plate at a density of 1 × 10^4^ cells per well and exposed to different concentrations of taxol (0.25 to 64,000 nM) for 2 days. Thereafter, a CCK‐8 reagent (10 μl; Dojindo) was added to each well and maintained at 37°C for another 2 h. The absorbance was measured at 450 nm under a microplate reader (Bio‐Rad). The half‐maximal inhibitory concentration (IC_50_) of taxol was obtained by using the Logit method.

### 2.8 Cell invasion and migration assay

The cell invasion and migration abilities were determined by using the Transwell chamber (Corning) pre‐coated with (invasion) or without (migration) matrigel. Briefly, cells were added into the upper chamber of 8 μm Transwell, and medium (600 μl) supplementing with 10% fetal bovine serum was added to the lower chamber as a chemoattractant. After 24‐h cultivation, cells adherent to the lower surface were fixed with methanol and acetone and stained with DAPI. Stained cells were photographed and counted under a microscope (Nikon).

### 2.9 Western blot

Western blot was conducted using the primary antibodies against Palladin (Cat No. 66601‐1‐Ig, Proteintech), Vimentin (Cat No. 3932; CST), N‐cadherin (Cat No. 13116; CST), E‐cadherin (Cat No. 20874‐1‐AP; Proteintech), CD44 (Cat No. 3570; CST), CD133 (Cat No. ab19898; Abcam), SOX2 (Cat No. 3579; CST), Nanog (Cat No. 3580; CST), OCT4 (Cat No. 2750; CST), Wnt3a (Cat No. 2391, CST), FZD3 (Cat No. ab217032, abcam), phospho‐LRP6^(Ser1490)^ (Cat No. 2568; CST), LRP6 (Cat No. 2560; CST), Phospho‐GSK‐3α/β^(Ser21/9)^ (Cat No. 9331; CST), GSK‐3α/β (Cat No. 5676; CST), β‐catenin (Cat No. 9562, CST), phospho‐β‐catenin^(Ser33/37/Thr41)^ (Cat No. 9561; CST), TCF3 (Cat No. 67140‐1‐Ig, Proteintech), LEF1 (Cat No. 28540‐1‐AP, Proteintech), c‐Myc (Cat No. 67447‐1‐Ig, Proteintech), and β‐actin (Cat No. 4970; CST). The goat anti‐mouse IgG or goat anti‐rabbit IgG or conjugated to horseradish peroxidase (HRP) were used as secondary antibodies. Visualization of the proteins was conducted with the chemiluminescence kit(Millipore).

### 2.10 Tumorigenicity assay

Specific‐pathogen‐free (SPF) female BALB/C nude mice (age, 4–5 weeks; weight, 14–16 g) were acquired from Huafukang Company. Mice were housed under a standard SPF condition (a 12‐h light/dark cycle, 25 ± 1°C, and 55% ± 5% humidity). The BALB/C nude mice were randomized into 6 groups (each group had 5 mice). The single‐cell suspension of various transfected cells (3 × 10^6^) was injected into the lateral thigh of the mice. After tumors reached the palpable size, the long and short diameters of the tumor were detected using an external caliper every 3 days after the inoculation. Tumor volume was calculated as (length×width^2^)/2. After 30 days, all mice were killed by cervical dislocation. Subsequently, tumors were harvested, photographed, and weighed. The harvested tumor tissues were fixed in the 4% paraformaldehyde, embedded into paraffin, and then prepared into 4 μm sections.

### 2.11 Immunohistochemistry (IHC)

The tissue microarray or sections were consecutively deparaffinized, rehydrated, and washed. After that, the antigen was repaired with citrate solution, and endogenous peroxidase activity was blocked with 3% H_2_O_2_ and goat serum. Primary antibody against Palladin (Cat No. MA5‐16141, Invitrogen), CD44 (Cat No. 3570; CST), CD133 (Cat No. ab19898; Abcam), Ki67 (Cat No. ab15580; Abcam), N‐cadherin (Cat No. 13116; CST), and β‐catenin (Cat No. 9562, CST), as well as biotinylated secondary antibody and HRP‐conjugated avidin, were used for incubation. The 3, 3′‐diaminobenzidine was used as the chromogen.

### 2.12 Statistical analysis

The data were analyzed by using SPSS software and GraphPad Prism 7.0. Data were expressed as the mean ± standard error of the mean (S.E.M) or number (percentage, %). Statistical significance between two groups was analyzed by chi‐square test or student's *t*‐test; among multiple groups, were analyzed by analysis of variance (ANOVA) with Bonferroni post hoc test. Survival analysis was estimated by the Kaplan–Meier method and compared by the log‐rank test. The relationship between Palladin and β‐catenin was verified by Spearman correlation analysis. Data were deemed statistically significant from a *p‐*value <0.05.

## 3 RESULTS

### 3.1 High expression of Palladin predicts a poor prognosis for patients with NSCLC

**FIGURE 1:**
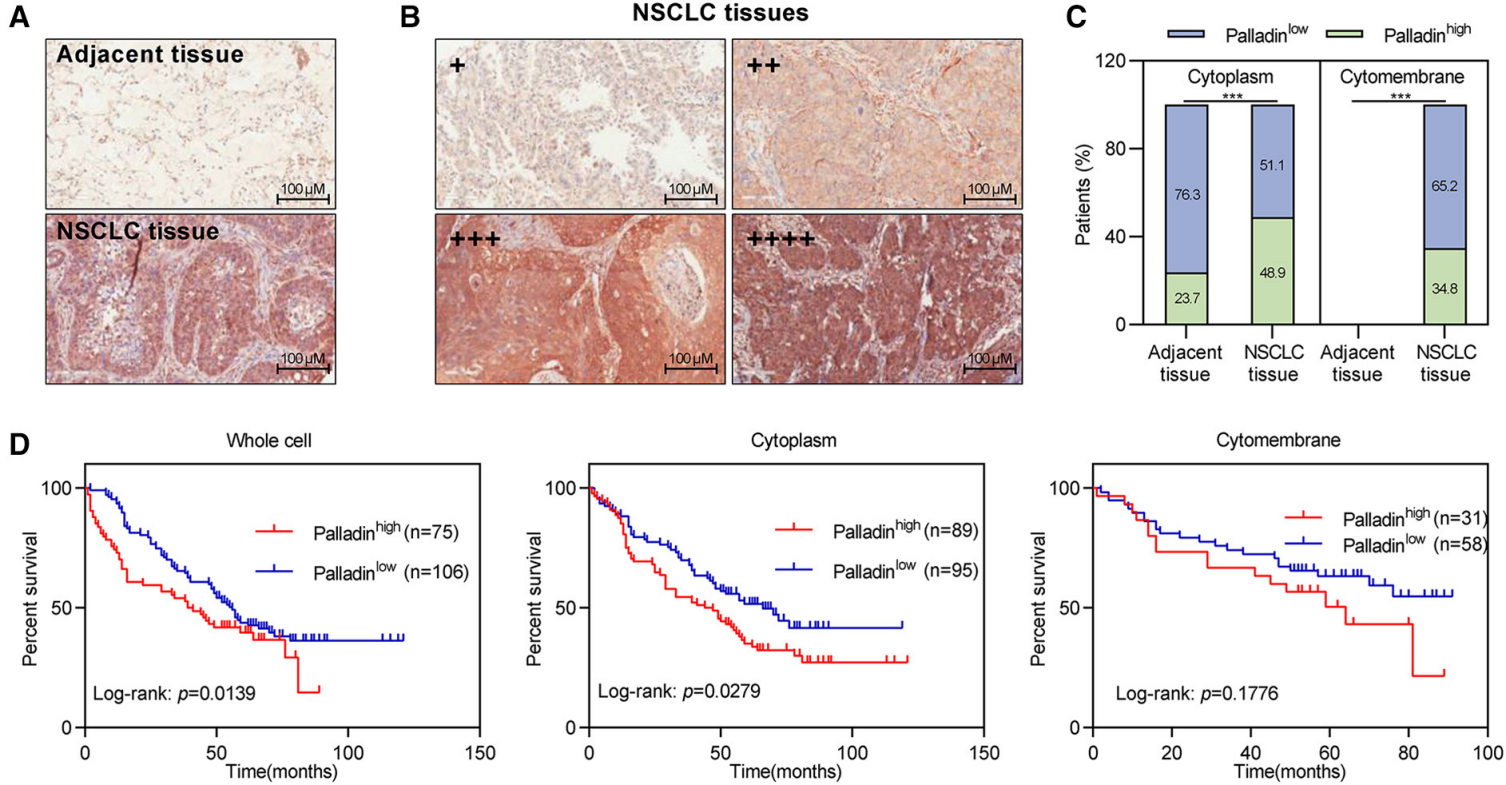
High expression of Palladin predicts a poor prognosis for patients with NSCLC. (A) Expression of Palladin in NSCLC and paracancerous tissues (scale bar, 100 μm). (B) According to IHC staining intensity and percentage of positively stained cells, Palladin expression was categorized into four grades: negative (−), weakly positive (+), positive (++), and strongly positive (+++) (scale bar, 100 μm). (C) The percentage of patients with Palladin^high^ or Palladin^low^ expression in the cytoplasm and cytomembrane. (D) Kaplan–Meier survival curve of overall survival according to Palladin expression in patients with NSCLC. Statistics: *p < 0.05, ***p < 0.001.

To explore the clinical role of Palladin in the disease progression of NSCLC, IHC staining was performed to determine whether any correlation between Palladin expression with survival outcomes in NSCLC samples. By using the tissue microarrays, the expression of Palladin was higher in NSCLC tissues compared to the paracancerous tissues (Figure 1A). According to the staining intensity and percentage of positively stained cells, the Palladin expression was categorized into four grades: negative (−), weakly positive (+), positive (++), and strongly positive (+++) (Figure 1B). Furthermore, the patients were stratified as either Palladin low expression (negative/weakly positive staining) or Palladin high expression (positive/strongly positive). Notably, Palladin was identified to be highly expressed in the cytoplasm and specifically expressed in the cytomembrane of patients with NSCLC (Figure 1C). Kaplan–Meier analysis showed that NSCLC patients with high Palladin expression (40 months) have shorter overall survival than patients with low SIPR1 expression (56 months) (Figure 1D). Meanwhile, Palladin expression in the cytoplasm is observed to be associated with the patients' survival (Figure 1D). Thus, these results implied that Palladin might be involved in the disease progression of NSCLC.

### 3.2 Enrichment and expression of Palladin in sphere cells of NSCLC

**FIGURE 2:**
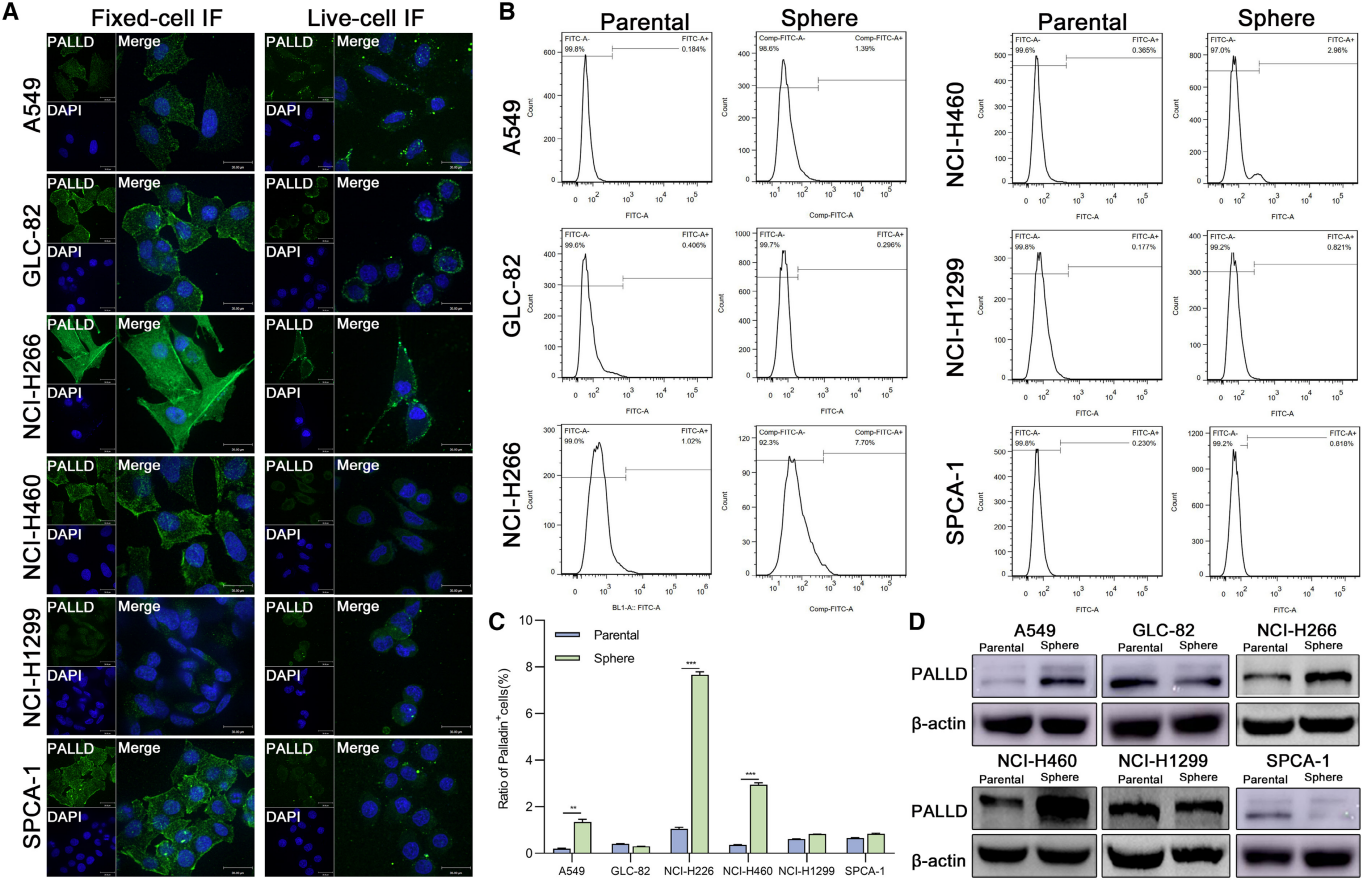
Enrichment and expression of Palladin in sphere cells of NSCLC. (A) Fixed‐cell and live‐cell immunofluorescence staining of Palladin in the NSCLC cell lines. (scale bar, 30 μm). (B, C) flow fluorescence assay detected the positive expression of Palladin in the parental and sphere cells of NSCLC cell lines. The quantitative results were shown. (D) Western blot measured the expression of Palladin in the parental and sphere cells of NSCLC cell lines. Data plots are expressed as mean ± S.E.M of three independent experiments. Statistics: *p < 0.05, **p < 0.01, ***p < 0.001.

To identify the localization and expression of Palladin, live‐cell and fixed‐cell immunofluorescence (IF) staining was conducted in six NSCLC cell lines, including A549, GLC‐82, NCI‐H1299, NCI‐H226, NCI‐H460, and SPCA‐1. IF staining demonstrated that Palladin was strongly expressed in cytoplasm and cytomembrane of NCI‐H226 and GLC‐82, but weakly in NCI‐H1299 and A549 (Figure 2A). We also observed that Palladin is widely expressed in the cytoplasm, membrane, and nucleus of NSCLC cell lines (Figure 2A), implying its potential for CSCs identification. Subsequently, we enriched CSCs by the serum‐free enrichment culture and detected the Palladin expression in parental and sphere cells by flow fluorescence assay and western blot. The results revealed that the Palladin^+^ cell percentage of sphere cells from A549, NCI‐H226, and NCI‐H460 was higher than that of the parental cells (Figure 2B,C). Consistent findings were observed in the western blotting (Figure 2D). Collectively, Palladin is widely expressed and enriched in the sphere cells of NSCLC.

Based on the expression levels of Palladin in various NSCLC cell lines, NCI‐H226 and NCI‐H460 with high endogenous expression, while A549 with low endogenous expression were selected for the subsequent experiments.

### 3.3 Palladin promoted stem cell‐like properties of NSCLC cells both in vitro and in vivo

**FIGURE 3:**
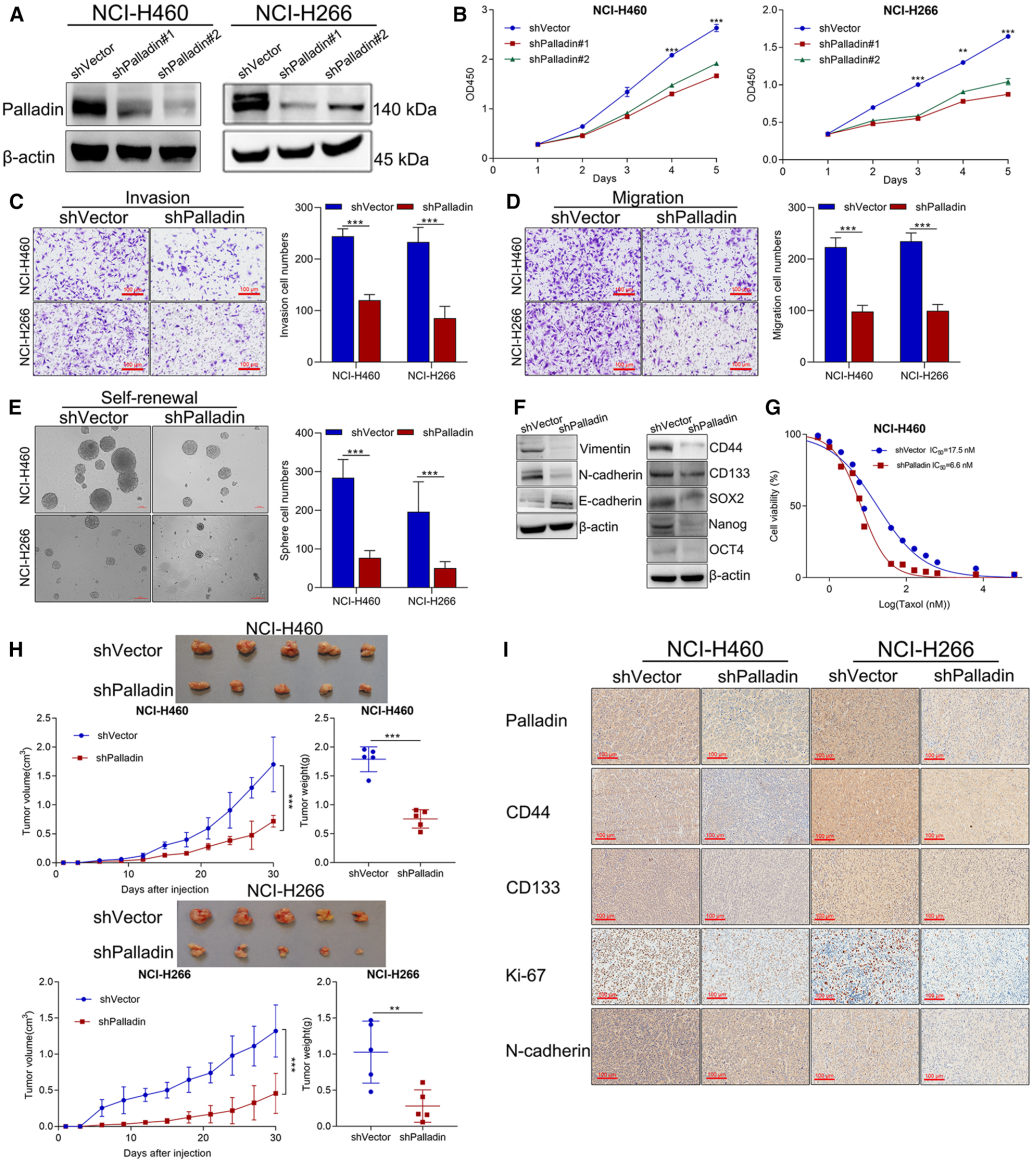
Knockdown of Palladin‐suppressed stem cell‐like properties of NSCLC cells both in vitro and in vivo. (A) NCI‐H460 and NCI‐H266 cells were transfected with lentivirus vector to knockdown Palladin. The protein expression of Palladin was detected by Western blot. (B) Cell vitality of transfected cells was detected by CCK‐8 assay. (C, D) Cell invasion and migration abilities of transfected cells were evaluated by the Transwell chamber. Representative images and quantitative data were shown (scale bar, 100 μm). (E) Self‐renewal ability of transfected NCI‐H460 and NCI‐H266 cells was evaluated by sphere formation assay. Representative images and quantitative data were shown. (G) Western blot of EMT and stemness markers. (F) Cell resistance to taxol was detected by the CCK‐8 assay. Data plots of in vitro experiments are expressed as mean ± S.E.M of three independent experiments. (H) Nude mice (*n* = 5 per group) were injected with the single‐cell suspension of transfected cells. After mice were killed, tumors were harvested and representative tumors, tumor growth curve, and weight were shown. (I) IHC staining of Palladin, CD44, CD133, Ki67, and N‐cadherin (scale bar, 100 μm). Statistics: *p < 0.05, **p < 0.01, ***p < 0.001.

To investigate the effect of Palladin on stem cell‐like properties of NSCLC cells, we established the Palladin‐knockdown stable cell line of NCI‐H226 and NCI‐H460 (Figure 3A). From the CCK‐8 assay, we found that the knockdown of Palladin suppressed the cell viability both in the NCI‐H226 and NCI‐H460 cells (Figure 3B). Besides, the invasion and migration abilities were significantly inhibited in Palladin‐knockdown cells compared to control cells (Figure 3C,D). Similarly, the Palladin knockdown significantly decreased the sphere‐forming ability in two cells (Figure 3E). Moreover, the expression of epithelial‐mesenchymal transition (EMT) markers (E‐cadherin, N‐cadherin, and Vimentin) and stemness markers (CD44, CD133, SOX2, Nanog, and OCT4) were measured using western blot after Palladin knockdown. As a result, N‐cadherin, Vimentin, CD44, CD133, SOX2, Nanog, and OCT4 were down‐regulated, while E‐cadherin was upregulated in Palladin‐knockdown cells (Figure 3F). These expression results further supported that Palladin affected the self‐renewal, invasion, and migration abilities of LCSCs. Furthermore, the cell resistance to taxol was evaluated by using the CCK‐8 assay. As shown in Figure 3G, NCI‐H460 cell lines with Palladin knockdown (IC_50_ = 6.6 μM) exhibited a decreased resistance to taxol compared with the control cells (IC_50_ = 17.5 μM).

Given that tumorigenicity is one crucial characteristic of CSCs, we constructed the xenograft mouse model to further provide in vivo evidence for Palladin in the progression of NSCLC. NCI‐H266 and NCI‐H460 cells with shPalladin transduction were injected into the mice. Figure 3H reveals that the tumor size and weight were significantly lower with those of shPalladin downregulation compared with the controls. Thereafter, the IHC staining confirmed that CD44, CD133, Ki67, and N‐cadherin were significantly downregulated along with the Palladin knockdown (Figure 3I), which was consistent with the findings from in vitro results.

**FIGURE 4:**
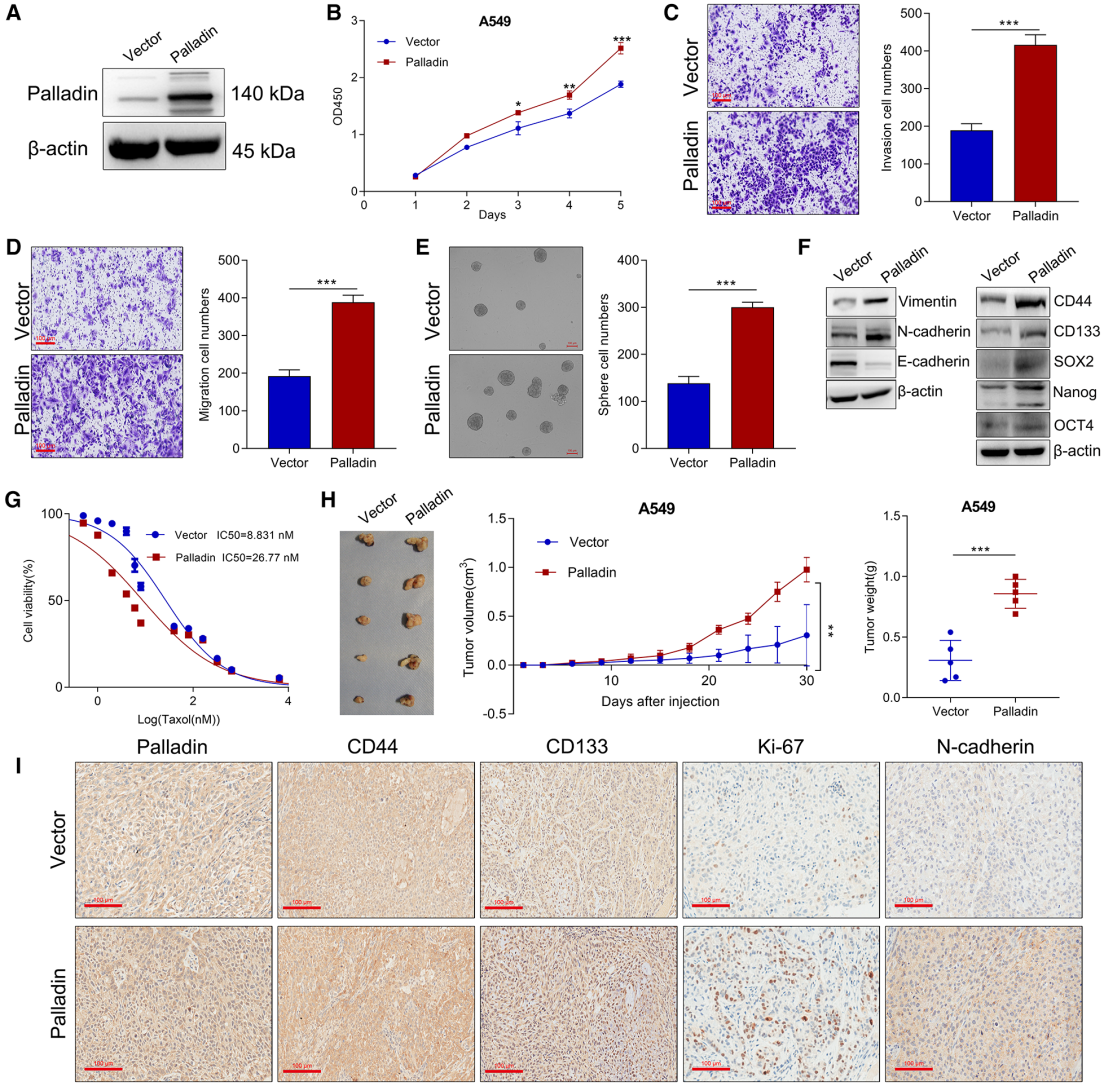
Overexpression of Palladin promoted stem cell‐like properties of NSCLC cells both in vitro and in vivo. (A) A5496 cell was transfected with lentivirus vector to upregulated Palladin. The protein expression of Palladin was detected by Western blot. (B) Cell vitality of transfected cells was detected by CCK‐8 assay. (C, D) Cell invasion and migration abilities of transfected cells were evaluated by the Transwell chamber. Representative images and quantitative data were shown (scale bar, 100 μm). (E) Self‐renewal ability of transfected cells was evaluated by sphere formation assay. Representative images and quantitative data were shown. (G) Western blot of EMT and stemness markers. (F) Cell resistance to taxol was detected by the CCK‐8 assay. Data plots of in vitro experiments are expressed as mean ± S.E.M of three independent experiments. (H) Nude mice (*n* = 5 per group) were injected with the single‐cell suspension of transfected A549 cells. After mice were killed, tumors were harvested and representative tumors, tumor growth curve, and weight were shown. (I) IHC staining of Palladin, CD44, CD133, Ki67, and N‐cadherin (scale bar, 100 μm). Statistics: *p < 0.05, **p < 0.01, ***p < 0.001.

In contrast, we established Palladin‐overexpressing cells in the A549 cells which had low endogenous expression (Figure 4A). After Palladin overexpression, we observed the opposite results to Palladin knockdown, that is, Palladin overexpression enhanced the cell vitality (Figure 4B), invasion (Figure 4C), migration (Figure 4D), and sphere‐forming ability (Figure 4E). Western blot also verified that Palladin overexpression enhanced the expression of N‐cadherin, Vimentin, CD44, CD133, SOX2, Nanog, and OCT4, but inhibited the E‐cadherin expression (Figure 4F). With regard to the cell resistance, the CCK‐8 assay (Figure 4G) confirmed that the resistance to taxol was significantly increased in the Palladin‐overexpressing cells (IC_50_ = 26.8 μM) than in the controls (IC_50_ = 8.8 μM). In the xenograft mouse model, the results showed that Palladin overexpression significantly promoted tumor growth with higher tumor size and weight than the controls (Figure 4H). Meanwhile, the IHC staining of CD44, CD133, Ki67, and N‐cadherin was found to be obviously suppressed after Palladin overexpression (Figure 4I). Taken together, these results revealed that Palladin promoted stem cell‐like properties of NSCLC cells both in vitro and in vivo.

### 3.4 Palladin regulates the stem phenotype of NSCLC cells by activating Wnt/β‐catenin signaling

**FIGURE 5:**
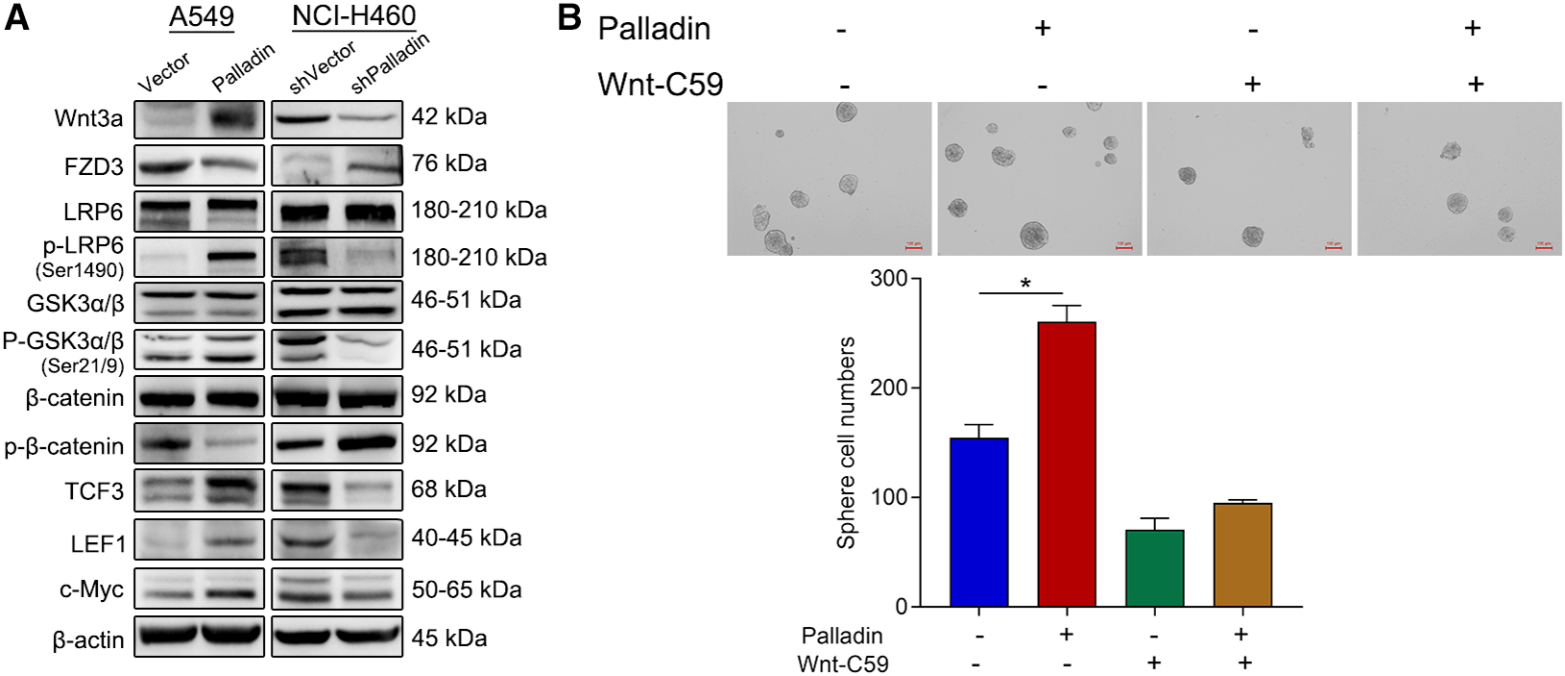
Palladin regulates the stem phenotype of NSCLC cells by activating Wnt/β‐catenin signaling. (A) Western blot of proteins in Wnt/β‐catenin signaling after Palladin overexpression or knockdown. (B) Analysis of the effect of Wnt/β‐catenin pathway inhibitor Wnt‐C59 on the self‐renewal ability of Palladin overexpressing cells and control cells (scale bar, 100 μm). Data plots are expressed as mean ± S.E.M of three independent experiments. Statistics: *p < 0.05.

To clarify the possible molecular mechanism of Palladin in the regulation of stem cell‐like properties, the expression of proteins in the Wnt/β‐catenin signaling that is essential for the maintenance of cancer stem cell‐like properties of cancer cells were analyzed. Western blot revealed that the overexpression of Palladin remarkably upregulated Wnt3a, p‐LRP6, p‐GSK‐3α, p‐GSK‐3β, TCF3, LEF1, and c‐Myc, but downregulated FZD3 and p‐β‐catenin (Figure 5A). However, the Palladin knockdown induced the opposite trends, that is, the expression of Wnt3a, p‐LRP6, p‐GSK‐3α, p‐GSK‐3β, TCF3, LEF1, and c‐Myc was suppressed while the expression of FZD3 and p‐β‐catenin was promoted (Figure 5A). By using the tissue microarrays, both Palladin and β‐catenin were highly expressed in NSCLC tissues, compared to paracancerous tissues (Table S1). Furthermore, the correlation analysis demonstrated a positive correlation between Palladin and β‐catenin (*R* = 0.51, *p* < 0.0001; Table S2). Thus, to further confirm the participation of Wnt/β‐catenin signaling, Wnt/β‐catenin pathway inhibitor Wnt‐C59 (10 μM) was added to the transfected cells. As indicated in Figure 5B, Wnt‐C59 blocked the Palladin‐induced enhancement of sphere‐forming. In summary, these data suggested that Palladin promotes cancer stem cell‐like properties by activating Wnt/β‐catenin signaling.

## 4 DISCUSSION

**FIGURE 6:**
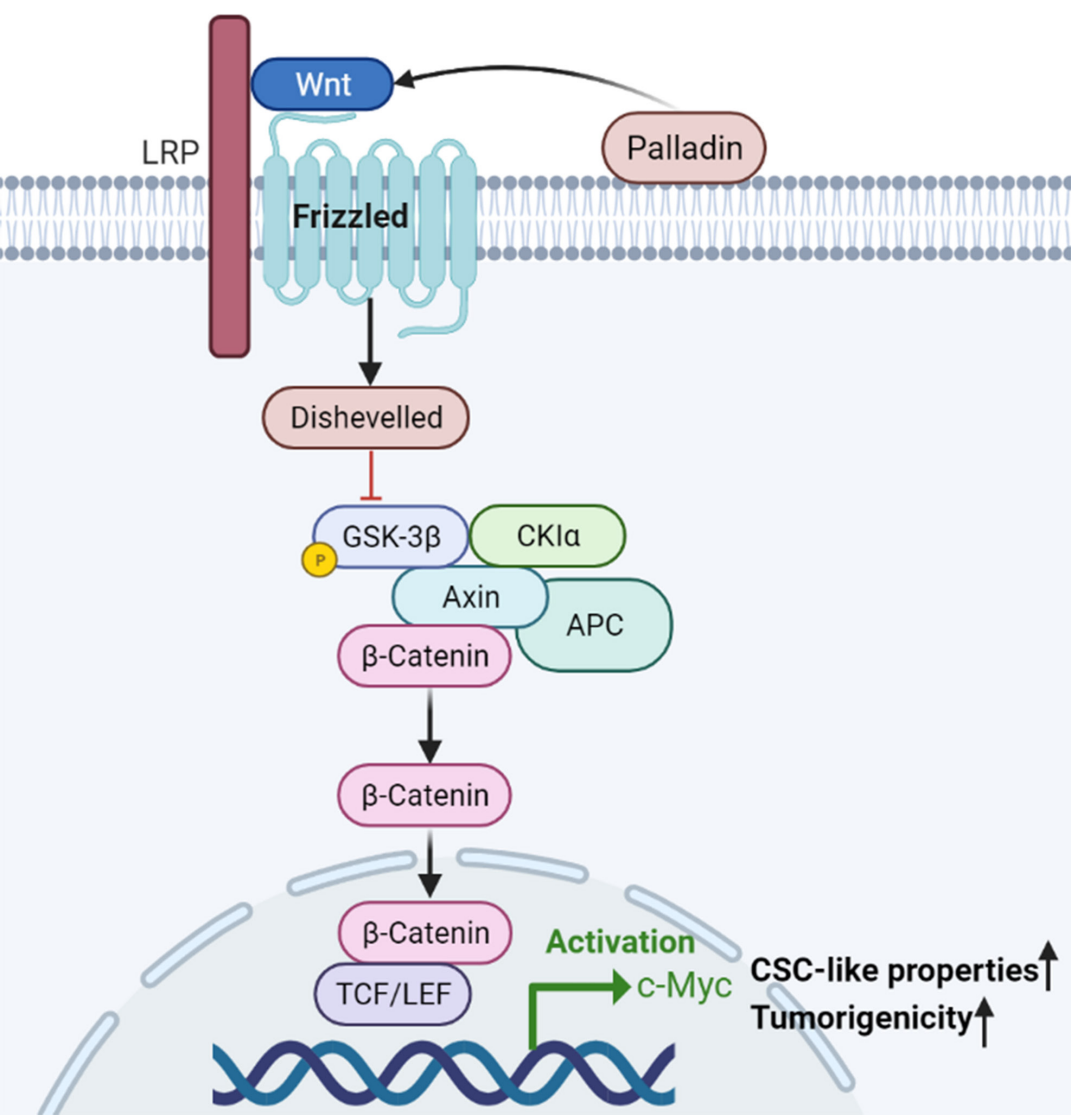
The potential molecular mechanism of Palladin in the NSCLC cells

Advances in CSC research might contribute to establishing successful therapies against tumors. Emerging evidence has proposed that selectively targeting specific surface markers and/or critical signaling of CSCs to repress the tumor progression can be exploited as an effective anti‐tumor therapeutic approach.^19^ However, until now, the clinical benefit of LCSC‐targeted therapies has not been demonstrated.^6^ Elaborating on the biological properties and regulation mechanisms of LCSCs is fundamental for the improvement of selective targeted therapy. Therefore, the present study preliminarily investigated the role of an actin‐associated protein Palladin and its potential molecular mechanism in the NSCLC (Figure 6).

Numerous studies have proved that actin‐associated proteins are accumulated in tumor cells and are involved in cell invasion and cancer progression.^20^ Among them, the actin‐associated protein Palladin was firstly reported in the cancer‐associated fibroblasts (CAFs) of pancreatic cancer.^16^ Subsequently, several studies confirmed the high expression of Palladin in multiple cancers^21^; however, the investigation of Palladin in lung cancer has not been yet described. Hereby, we measured the differential expression of Palladin between NSCLC and normal tissues using clinical samples. As expected, patients with NSCLC presented with a high level of Palladin expression, which is identified with other tumor types.^16^, ^21^, ^22^, ^23^ Consistent with the previous analysis,^22^, ^24^ the Kaplan–Meier analysis revealed that the elevated expression of palladin predicted the poor survival of patients. Bases on these results, it is evident that Palladin participated in the disease progression of NSCLC.

Nevertheless, the understanding of Palladin in cancers still formed at the level of normal cells rather than CSCs; thus, further research on its effects on CSCs is still needed. In the present study, we validated the elevated expression of Palladin in various NSCLC sphere cells. Interestingly, Palladin was identified to be specifically expressed in the cytomembrane of tumor cells, suggesting that Palladin might be applicable as a cell surface marker for LCSCs identification. Through in vitro and in vivo experiments, Palladin‐overexpressed NSCLC cell lines demonstrated the higher capability of spheroid colony formation in serum‐free media, invasion, and migration, as well as the higher ability to form xenograft tumors in BALB/C nude mice, consistent with the previous findings.^12^, ^15^, ^25^ We also analyzed the expression of EMT‐related (E‐cadherin, N‐cadherin, and Vimentin)^26^ and stem cell‐related genes (CD44, CD133, SOX2, Nanog, and OCT4).^27^ As a result, Palladin negatively regulated N‐cadherin, Vimentin, CD44, CD133, SOX2, Nanog, and OCT4, but positively regulated the E‐cadherin, which provides supportive evidence for phenotypic study. Meanwhile, the Palladin‐knockdown cell lines presented the opposite results. It is worth noting that the drug resistance ability of NSCLC cell lines was significantly affected by Palladin. As we know, the self‐renewal, tumorigenic properties, and drug resistance of CSCs may be responsible for the attempt failure of the CSCs‐targeted strategy.^28^ Here, it is evident that targeting Palladin could inhibit these key properties of CSCs; thereby, targeting Palladin may represent a potentially useful approach in invasive lung cancer.

Understanding downstream signaling of oncogenesis is pivotal to elucidating the pathogenic mechanisms of tumor progression and improving the therapeutic effect in targeted therapy. Despite the several studies that have revealed that Palladin promotes tumor cell invasion by regulating the activity of Cdc42,^16^ its specific mechanisms for regulating stem‐cell properties have never been reported previously. It has been widely accepted that several key developmental signaling pathways, such as the Notch, Wnt, Hedgehog, and Hippo signaling cascades, are crucial for stem cell homeostasis and function in various solid tumors.^18^ Of these, due to the strong conservation of Wnt signaling, it plays a key role in regulating the differentiation and pluripotency of stem cells.^29^ Thereby, we have focused on whether Palladin regulates the Wnt/β‐catenin signaling, which is the most intensively studied and best characterized Wnt signaling pathway.^30^ Through the western blot analysis, we observed that Palladin positively regulated the expression of Wnt3a, the inhibition of FZD3, as well as the phosphorylation and activation of the LRP6 receptor, which in turn induced the phosphorylation of GSK‐3α^Ser21^ and GSK‐3β^Ser9^. Then, the GSK3‐dependent phosphorylation of β‐catenin was suppressed and the nuclear accumulation of β‐catenin was increased. Thereafter, the downstream DNA‐binding proteins (i.e., TCF3 and LEF1) and targets, such as c‐Myc, were upregulated. The cascading changes of these proteins are consistent with the classical Wnt/β‐catenin signaling.^31^, ^32^ In addition, the results showed that the addition of Wnt‐C59 (a Wnt/β‐catenin pathway inhibitor) blocked the Palladin‐induced enhancement of sphere‐forming, further supporting the role of the Wnt/β‐catenin pathway as targeted downstream signaling of Palladin. Further analysis in NSCLC tissues also showed that Palladin was positively correlated with β‐catenin, which provided the in vivo evidence for this finding. In spite of no direct evidence on the relationship between Palladin and the Wnt/β‐catenin signaling pathway, the association of Palladin and Wnt/β‐catenin signaling has been implied in a previous study, that they were simultaneously up‐regulated by Sox9 to control self‐renewal and invasion in basal cell carcinoma.^33^ Similarly, other members of the actin‐associated proteins family were reported to play an oncogenic role via Wnt/β‐catenin signaling,^34^ as seen in our study. In summary, we assumed the potential mechanism that Palladin activates the Wnt/β‐catenin signaling by reducing the β‐catenin degradation and increasing the nuclear accumulation of β‐catenin to promote the stemness properties of NSCLC cells.

In conclusion, the present study firstly confirmed the overexpression of Palladin in both the tissues and sphere cells of lung cancers. Palladin was identified to be specifically expressed in the cytomembrane of patients with NSCLC, suggesting its potential as a cell surface marker for LCSCs identification. More importantly, we demonstrated that Palladin might serve as a tumor oncogene and promote the biological and stemness properties of NSCLC cells, possibly by activating the Wnt/β‐catenin signaling for the first time. The elucidation of this new molecular mechanism for Palladin and Wnt/β‐catenin signaling activation is conducive to understanding the pathogenesis of NSCLC, providing a novel possibility for the development of a novel therapeutic target for CSCs targeted therapy.

## AUTHOR CONTRIBUTIONS

**Xiong Shu:** Data curation (equal); formal analysis (equal); methodology (lead); validation (equal); writing – original draft (lead). **Meng Chen:** Data curation (equal); formal analysis (equal). **Shi‐Ya Liu:** Data curation (equal); formal analysis (equal); validation (equal). **Long Yu:** Resources (lead). **Li‐Xin Sun:** Project administration (equal); resources (equal). **Li‐Chao Sun:** Project administration (equal). **Yu‐Liang Ran:** Conceptualization (equal); writing – review and editing (lead).

## FUNDING INFORMATION

This research was supported by National Natural Science Foundation of China (Grant Number: 82073278); Beijing Natural Science Foundation (Grant Number: 7222012); Beijing Municipal Health Commission (Grant Number: BMHC‐2019‐9); Beijing Municipal Health Commission (Grant Number: BMHC‐2021‐6); the Independent Issue of State Key Laboratory of Molecular Oncology (Grant Number: SKL‐2019‐17).

## CONFLICT OF INTEREST

The authors declare that they have no conflict of interest.

## ETHICAL APPROVAL STATEMENT

The animal protocols were approved by Ethics Committee of Chinese Academy of Medical Sciences and Peking Union Medical College.

## Supporting information


Table S1
Table S2

## Data Availability

The data that support the findings of this study are available from the corresponding author upon reasonable request.

## References

[1] Reya T , Morrison SJ , Clarke MF , Weissman IL . Stem cells, cancer, and cancer stem cells. Nature. 2001;414(6859):105‐111. 11689955 10.1038/35102167

[2] Najafi M , Farhood B , Mortezaee K . Cancer stem cells (CSCs) in cancer progression and therapy. J Cell Physiol. 2019;234(6):8381‐8395. 30417375 10.1002/jcp.27740

[3] Phi LTH , Sari IN , Yang YG , et al. Cancer stem cells (CSCs) in drug resistance and their therapeutic implications in cancer treatment. Stem Cells Int. 2018;2018:5416923. 29681949 10.1155/2018/5416923 PMC5850899

[4] Batlle E , Clevers H . Cancer stem cells revisited. Nat Med. 2017;23(10):1124‐1134. 28985214 10.1038/nm.4409

[5] Barta JA , Powell CA , Wisnivesky JP . Global epidemiology of lung cancer. Ann Glob Health. 2019;85(1):8. 30741509 10.5334/aogh.2419 PMC6724220

[6] Heng WS , Gosens R , Kruyt FAE . Lung cancer stem cells: origin, features, maintenance mechanisms and therapeutic targeting. Biochem Pharmacol. 2019;160:121‐133. 30557553 10.1016/j.bcp.2018.12.010

[7] Prabavathy D , Swarnalatha Y , Ramadoss N . Lung cancer stem cells‐origin, characteristics and therapy. Stem Cell Investig. 2018;5:6. 10.21037/sci.2018.02.01 PMC5897668 29682513

[8] Zakaria N , Satar NA , Abu Halim NH , et al. Targeting lung cancer stem cells: research and clinical impacts. Front Oncol. 2017;7:80. 28529925 10.3389/fonc.2017.00080 PMC5418222

[9] Zhou HM , Zhang JG , Zhang X , Li Q . Targeting cancer stem cells for reversing therapy resistance: mechanism, signaling, and prospective agents. Signal Transduct Target Ther. 2021;6(1):62. 33589595 10.1038/s41392-020-00430-1 PMC7884707

[10] Henkin RI . Clinical and therapeutic implications of cancer stem cells. N Engl J Med. 2019;381(10):e19. 10.1056/NEJMc1908886 31483981

[11] Mykkänen OM , Grönholm M , Rönty M , et al. Characterization of human palladin, a microfilament‐associated protein. Mol Biol Cell. 2001;12(10):3060‐3073. 11598191 10.1091/mbc.12.10.3060 PMC60155

[12] Gilam A , Conde J , Weissglas‐Volkov D , et al. Local microRNA delivery targets Palladin and prevents metastatic breast cancer. Nat Commun. 2016;7:12868. 27641360 10.1038/ncomms12868 PMC5031803

[13] Najm P , El‐Sibai M . Palladin regulation of the Actin structures needed for cancer invasion. Cell Adh Migr. 2014;8(1):29‐35. 24525547 10.4161/cam.28024 PMC3974790

[14] Albraiki SE . The Role of the 90 kDa Palladin in the Regulation of Actin Filaments. Wichita State University; 2020.

[15] Chin YR , Toker A . The Actin‐bundling protein palladin is an Akt1‐specific substrate that regulates breast cancer cell migration. Mol Cell. 2010;38(3):333‐344. 20471940 10.1016/j.molcel.2010.02.031 PMC2872630

[16] Goicoechea SM , García‐Mata R , Staub J , et al. Palladin promotes invasion of pancreatic cancer cells by enhancing invadopodia formation in cancer‐associated fibroblasts. Oncogene. 2014;33(10):1265‐1273. 23524582 10.1038/onc.2013.68 PMC3912215

[17] Li F , Tiede B , Massagué J , Kang Y . Beyond tumorigenesis: cancer stem cells in metastasis. Cell Res. 2007;17(1):3‐14. 17179981 10.1038/sj.cr.7310118

[18] Clara JA , Monge C , Yang Y , Takebe N . Targeting signalling pathways and the immune microenvironment of cancer stem cells ‐ a clinical update. Nat Rev Clin Oncol. 2020;17(4):204‐232. 31792354 10.1038/s41571-019-0293-2

[19] Pan Y , Ma S , Cao K , et al. Therapeutic approaches targeting cancer stem cells. J Cancer Res Ther. 2018;14(7):1469‐1475. 30589025 10.4103/jcrt.JCRT_976_17

[20] Izdebska M , Zielinska W , Grzanka D , Gagat M . The role of Actin dynamics and Actin‐binding proteins expression in epithelial‐to‐mesenchymal transition and its association with cancer progression and evaluation of possible therapeutic targets. Biomed Res Int. 2018;2018:4578373. 29581975 10.1155/2018/4578373 PMC5822767

[21] Goicoechea SM , Bednarski B , García‐Mata R , Prentice‐Dunn H , Kim HJ , Otey CA . Palladin contributes to invasive motility in human breast cancer cells. Oncogene. 2009;28(4):587‐598. 18978809 10.1038/onc.2008.408 PMC2633435

[22] Gupta V , Bassi DE , Simons JD , et al. Elevated expression of stromal Palladin predicts poor clinical outcome in renal cell carcinoma. PLOS One. 2011;6(6):e21494. 21738681 10.1371/journal.pone.0021494 PMC3125241

[23] Davidson B , Bock AJ , Holth A , Nymoen DA . Expression of palladin is associated with disease progression in metastatic high‐grade serous carcinoma. Cytopathology. 2020;31(6):572‐578. 32741023 10.1111/cyt.12895

[24] Sato D , Tsuchikawa T , Mitsuhashi T , et al. Stromal Palladin expression is an independent prognostic factor in pancreatic ductal adenocarcinoma. PLOS One. 2016;11(3):e0152523. 27023252 10.1371/journal.pone.0152523 PMC4811423

[25] Brentnall TA , Lai LA , Coleman J , Bronner MP , Pan S , Chen R . Arousal of cancer‐associated stroma: overexpression of palladin activates fibroblasts to promote tumor invasion. PLoS One. 2012;7(1):e30219. 22291919 10.1371/journal.pone.0030219 PMC3264580

[26] Kaszak I , Witkowska‐Pilaszewicz O , Niewiadomska Z , Dworecka‐Kaszak B , Ngosa Toka F , Jurka P . Role of cadherins in cancer‐a review. Int J Mol Sci. 2020;21(20):7624. 33076339 10.3390/ijms21207624 PMC7589192

[27] Baillie R , Tan ST , Itinteang T . Cancer stem cells in oral cavity squamous cell carcinoma: a review. Front Oncol. 2017;7(112):112. 28626726 10.3389/fonc.2017.00112 PMC5454033

[28] Eramo A , Haas TL , De Maria R . Lung cancer stem cells: tools and targets to fight lung cancer. Oncogene. 2010;29(33):4625‐4635. 20531299 10.1038/onc.2010.207

[29] Valkenburg KC , Graveel CR , Zylstra‐Diegel CR , Zhong Z , Williams BO . Wnt/β‐catenin signaling in normal and cancer stem cells. Cancer. 2011;3(2):2050‐2079. 10.3390/cancers3022050 PMC3757404 24212796

[30] Takahashi‐Yanaga F , Kahn M . Targeting Wnt signaling: can we safely eradicate cancer stem cells? Clin Cancer Res. 2010;16(12):3153‐3162. 20530697 10.1158/1078-0432.CCR-09-2943

[31] Holland JD , Klaus A , Garratt AN , Birchmeier W . Wnt signaling in stem and cancer stem cells. Curr Opin Cell Biol. 2013;25(2):254‐264. 23347562 10.1016/j.ceb.2013.01.004

[32] Zhang Y , Wang X . Targeting the Wnt/beta‐catenin signaling pathway in cancer. J Hematol Oncol. 2020;13(1):165. 33276800 10.1186/s13045-020-00990-3 PMC7716495

[33] Larsimont JC , Youssef KK , Sanchez‐Danes A , et al. Sox9 controls self‐renewal of oncogene targeted cells and links tumor initiation and invasion. Cell Stem Cell. 2015;17(1):60‐73. 26095047 10.1016/j.stem.2015.05.008

[34] Lian G , Dettenhofer M , Lu J , et al. Filamin A‐ and formin 2‐dependent endocytosis regulates proliferation via the canonical Wnt pathway. Development. 2016;143(23):4509‐4520. 27789627 10.1242/dev.139295 PMC5201043

