# Supplementary material for: Palladin promotes cancer stem cell‐like properties in lung cancer by activating Wnt/Β‐Catenin signaling
Source: Cancer Med. 2022 Sep 1;12(4):4510–20. doi: 10.1002/cam4.5192 (PMC9972019; doi:10.1002/cam4.5192)
Supplement: Supplementary file 1 — Table S1 Table S2 [file CAM4-12-4510-s001.docx]

Table S1. Percentage of patients with Palladin/β-catenin positive or negative expression according to tissue microarrays analysis.

|  | NSCLC tissues | Paracancerous tissues | P value |
| --- | --- | --- | --- |
| Palladin positive | 72 (76.60) | 7 (8.24) | <0.0001 |
| β-catenin positive | 74 (78.72) | 0 | <0.0001 |

Table S2. Correlation analysis between Palladin and β-catenin according to tissue microarrays analysis.

| Palladin | β-catenin | | R | *P* value |
| --- | --- | --- | --- | --- |
|  | Positive | Negative |  |  |
| Positive | 65 | 14 | 0.5107 | <0.0001 |
| Negative | 9 | 91 |  |  |

* R, correlation coefficient.
